# Characteristics of depressive symptoms in middle-aged family members of dementia patients: 2017 Korea Community Health Survey

**DOI:** 10.4178/epih.e2020031

**Published:** 2020-05-15

**Authors:** Jinbeom Park, Won-Chul Lee, Hyunsuk Jeong, Nayoung Hong, BoYoung Bae, Hyeon Woo Yim

**Affiliations:** Department of Preventive Medicine, College of Medicine, The Catholic University of Korea, Seoul, Korea

**Keywords:** Family with dementia patient, Depressive symptom, PHQ-9, Korea Community Health Survey

## Abstract

**OBJECTIVES:**

The characteristics of depressive symptoms in the family members of home-dwelling patients with dementia have not been clearly reported. This study aimed to investigate the characteristics of depressive symptoms in middle-aged family members living with a patient with dementia.

**METHODS:**

This study used the data from the nationwide 2017 Korea Community Health Survey. Among the 228,381 survey participants, 77,276 participants in their 40s and 50s were finally selected for this study. The participants consisted of 760 family members of home-dwelling dementia patients and 76,516 general family members comprising a control group.

**RESULTS:**

The positive rate of Patient Health Questionnaire-9 (PHQ-9)-measured depressive symptoms was significantly higher in the family members of home-dwelling dementia patients (4.4%; control group: 1.9%). After adjusting for potential confounders, the prevalence of PHQ-measured depressive symptoms was 1.72 times (95% confidence interval [CI], 1.03 to 2.85) higher in the family members of home-dwelling dementia patients compared to the control group. The positive rate of depressive symptoms was 2.26 times higher (95% CI, 1.26 to 4.05) in the female middle-aged family members of home-dwelling dementia patients compared to the control group. In addition, those who reported having symptoms almost every day in the PHQ-9 questions had significantly higher positive rates on questions about loss of interest, depression, sleep disturbance, fatigue, poor appetite, and suicidal ideation, and not on questions regarding feelings of worthlessness and psychomotor agitation, compared to the control group.

**CONCLUSIONS:**

Active interventions are needed to relieve depression in the family members of home-dwelling dementia patients.

## INTRODUCTION

Dementia is a fatal disease that affects over 46.8 million people worldwide. This figure is expected to double every 20 years, reaching 75 million by 2030 and approximately 130 million by 2050 [1]. Looking at the domestic status, the estimated number of patients with dementia in Korea in 2017 was 702,436 out of the total 7,066,201 elderly people across the country, and the number of family members of patients with dementia, including spouses, children, and grandchildren was approximately 3.5 million [2].

As a dementia-related policy in Korea, the National Responsibility System for Dementia was implemented in 2017, and started providing various services for patients with dementia and their family members. Although various programs such as self-help groups and vocational programs for family caregivers of patients with dementia are being implemented, their effect on alleviating care burden among family caregivers is insignificant [3].

Among the family members of home-dwelling patients with dementia, sons and their wives (patients’ daughters-in-law) often care for home-dwelling patients with dementia, and 50-60% of family members living together are middle-aged, in their 40s and 50s, who are engaged in economic activities and bearing the care burden of the patients with dementia [4]. Consequently, they experience economic burden due to limited economic activities and dementia-related medical expenses [5].

The family members of patients with dementia have been reported to play a central role in caring for the patients, and as patients’ cognitive impairment deteriorates, family caregivers’ physical and mental efforts required for continuous care increase, thereby increasing the risk of stress and depression [6]. In addition, caregiver burden for caring for elderly persons with dementia was reported to be higher than for caring for elderly persons with chronic diseases or stroke [7].

Depressive symptoms are the most common psychiatric symptoms experienced by the caregivers of patients with dementia, and 75% of caregivers of elderly persons with dementia were reported to experience depressive symptoms [8]. In addition, approximately 60% of family caregivers of patient with dementia were found to have sleep disturbance [9], and 16% had contemplated suicide more than once [10]. Furthermore, they had poor health status [5,11] and were at a high risk of stroke [12]. Although it has been reported that family members of patients with dementia have various mental burdens including depression as well as physical burdens, the characteristics of depressive symptoms have not been clearly reported. Therefore, this study aims to compare the prevalence of Patient Health Questionnaire-9 (PHQ-9)-measured depressive symptoms and the characteristics of depressive symptoms for each PHQ-9 question, and to determine the characteristics of depressive symptoms in family members of home-dwelling patients with dementia using the data from the nationally representative 2017 Korea Community Health Survey (KCHS).

## MATERIALS AND METHODS

### Study design

This is a cross-sectional study aimed to determine the prevalence of depression and the characteristics of depressive symptoms for each PHQ-9 question in middle-aged families of patients with dementia using the data from the KCHS that was conducted nationwide in 2017.

### Participants and data collection

Since 2008, the KCHS is conducted annually in accordance with the Community Health Act, with the target population of adults aged 19 years or older as of July 1st of the respective year [13].

One-person households that had no possibility of caring for a patient with dementia were excluded. In addition, considering the fact that those in their 20s and 30s are unlikely to be family caregivers of patients with dementia, and that dementia is predominant in the elderly population (i.e., persons aged 65 years or older), middle-aged persons (or those in 40s and 50s) were selected as the children generation of dementia patients for this study.

**Figure 1.**
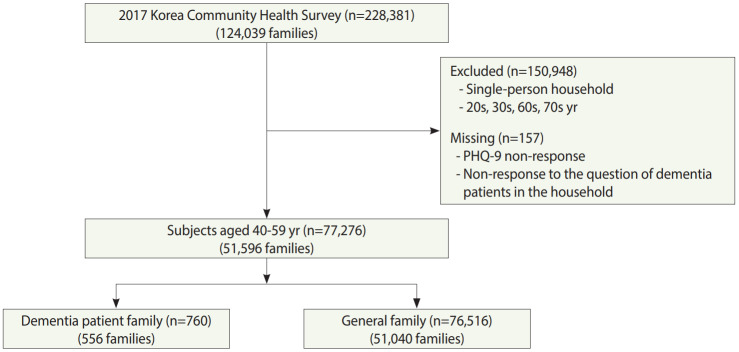
Flow chart of this study. PHQ-9, Patient Health Questionnaire-9.

Therefore, among 228,381 survey participants in 2017, excluding single-person households, and selecting those in their 40s and 50s, and further excluding three who did not respond to the question regarding whether or not they were living with a patient with dementia, and 157 who did not respond to the PHQ-9 questions, a total of 77,276 participants were finally selected. Families who were currently living with a patient diagnosed with dementia by a physician were defined as family members of home-dwelling patients with dementia, and families not living with a patient with dementia were defined as general families comprising the control group. The sample consisted of 760 family members of home-dwelling dementia patients, and 76,516 general family members in the control group (Figure 1).

### Instruments

#### General characteristics

The information of gender, age, household type, household income, employment status, education level, residential area, current smokers, drinking alcohol, physical activity (PA), and body mass index (BMI) were collected from the survey. Age was divided into those in their 40s (40-49 years old), and those in their 50s (50-59 years old); employment was divided into employed (those with a job), student/repeater, housewife, and unemployed (those without a job/vocation); the household type was classified as one, two, and three generational households; practicing moderate PA for more than 30 minutes a day for more than five days per week, or practicing vigorous PA for more than 20 minutes a day for more than three days per week during the one week preceding data collection was considered as moderate or high PA.

#### Depressive symptoms and depressive mood

Depressive symptoms were assessed using the PHQ-9. The PHQ-9 is widely used as a screening tool for major depressive disorders, and is known to have high sensitivity (88%) and specificity (88%) [14]. The PHQ-9 consists of nine questions. Each question is scored from 0 to 3, and the total score ranges from 0 to 27. In this study, the criteria for major depressive disorder, that is, a PHQ-9 score of ≥ 10 points was used as the cut-off point. To identify the characteristics of depression, a score of 0-2 points for each question in the PHQ-9 was reclassified as negative, and a score of 3 points was reclassified as positive to determine the response rate. The Cronbach’s alpha reliability of the scale was 0.78 in this study.

Those who felt sadness or hopelessness interfering with their daily lives for more than two consecutive weeks in the one year preceding data collection were considered as experiencing depressive mood.

### Statistical analysis

The statistical analysis was performed using the SAS version 9.4 (SAS Institute Inc., Cary, NC, USA), and the data were analyzed using the complex sample design method considering the weight. The statistical significance level was set at p-value < 0.05.

In order to compare the characteristics of the participants in the two groups according to households with or without homed-welling patients with dementia, the participants’ characteristics were presented as frequencies and percentages, and the means and standard errors for the continuous variables were presented.

The comparison of the differences between the two groups was performed for categorical variables using the Rao-Scott chi-square test, and for continuous variables using the t-test.

The differences between the prevalence of PHQ-9 measured depressive symptoms and the positive rate of depressive symptoms for each PHQ-9 question was determined using the RaoScott chi-square test.

Confounding variables included demographic variables, and income level and economic activity variables, which are known as burden factors [15] in the family members of home-dwelling patients with dementia. Monthly alcohol drinking with a p-value of ≤ 0.25 found in the univariate analysis, and current smoking were used as adjustment variables. Multivariable logistic regression analysis was performed to determine the relationship between the presence or absence of home-dwelling dementia patient and depressive symptoms in family members of patients with dementia.

### Ethics statement

This study was approved by the Institutional Review Board of The Catholic University of Korea (approval No. MC19ZESI0051).

## RESULTS

### The general characteristics of family members of home-dwelling patients with dementia and general family members in the control group

The results of a comparison of the general characteristics of the two groups showed significant differences in age, household income, economic activity, education level, household by generation, residential area, current smoking, monthly alcohol drinking, and number of diagnosed diseases (p<0.05). By age group, the proportion of family members aged 50-59 years of home-dwelling dementia patients was 60.2%, and the proportion of those aged 40-49 years was 39.8%. In the control group, the proportion of those aged 40-49 years was 51.3%, and the proportion of those aged 50-59 years was 48.7%.

The proportion of those with a household income < 1 million Korean won (KRW) was higher in the families of home-dwelling dementia patients (11.0%) than in the general families in the control group (2.9%), whereas the proportion of those with a household income ≥ 3 million KRW was higher in the control group (74.5%) than in the families of home-dwelling dementia patients (51.9%). The proportion of those who were unemployed was higher in the families of home-dwelling dementia patients (25.3%) than in the general families in the control group (20.5%). The proportion of current smokers was higher in the families of home-dwelling dementia patients (26.9%) than in the control group (22.4%). The proportion of those with monthly drinking was higher in the control group (58.0%) than in the families of home-dwelling dementia patients (63.7%) (Table 1).

### Group differences on depression

The rate of experience of depression as measured with a single question was higher in the families of home-dwelling dementia patients (12.2%) than in the control group (5.6%) (Table 2). The prevalence of depressive symptoms measured by the PHQ-9 was higher in the families of home-dwelling dementia patients (4.4%) than in the control group (1.9%). In terms of gender, the prevalence of depressive symptoms in men was higher in the families of home-dwelling dementia patients (3.2%) than in the control group (1.5%), and also higher in women in the families of homed-welling dementia patients (5.9%) than in the control group (2.2%).

The prevalence of depressive symptoms was significantly higher in the family of home-dwelling dementia patients with an odds ratio (OR) of 2.46 (95% confidence interval [CI], 1.56 to 3.88) and an adjusted odds ratio (aOR) of 1.72 (95% CI, 1.03 to 2.85) compared to the control group after adjusting for potential confounders. Sensitivity analysis was performed with different selection criteria that were limited to 40s and 50s. Similar results were obtained by changing the age category into 30-69 year and 40-69 years (Supplementary Materials 1 and 2).

The crude OR and aOR were significantly higher at 2.75 (95% CI, 1.61 to 4.68) and 2.26 (95% CI, 1.26 to 4.05), respectively, in the female family members of home-dwelling dementia patients, compared to female family members in the control group. The difference was not significant in the male family members in the two groups (Table 3).

### Characteristics of families of home-dwelling dementia patients and control families according to each Patient Health Questionnaire-9 question

**Figure 2.**
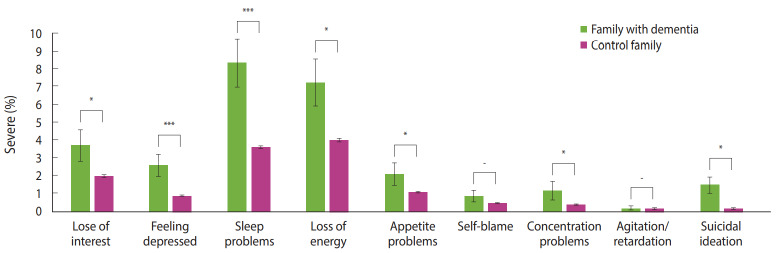
Proportion of severe answered Patient Health Questionnaire-9 item between family with dementia patients at home and control family. ^*^p<0.05, ^***^p<0.001.

The analysis of differences between the two groups according to each PHQ-9 question showed that the positive rates of loss of interest, depression, sleep disturbance, fatigue, and appetite problems were higher in the family members of home-dwelling dementia patients. Moreover, the positive rate of thinking about death almost every day was also higher in this group (1.5%) than in the control group (0.2%). There was no difference in two areas such as feelings of worthlessness and psychomotor (p = 0.064, p= 0.905, respectively) (Figure 2, Table 4).

## DISCUSSION

This study investigated the difference in the positive rate of the PHQ-9 measured depressive symptoms between the middle-aged family members of home-dwelling dementia patients and the middle-aged family members in the control group, and investigated the positive rate of depressive symptoms for each PHQ-9 question to identify the characteristics of the family members of home-dwelling dementia patients using the data from the 2017 KCHS.

In this study, the mean age of the middle-aged family members of home-dwelling dementia patients was 51 years, which was consistent with a previous study by Kim et al. [16] indicating that the mean age of the main caregivers of dementia patients was 52.3 years old, and that most of the main caregivers were middle-aged.

Household income and economic activity were significantly lower in the family members of home-dwelling dementia patients. A study by Choi et al. [17], reported that 27% of the caregivers of dementia patients quit their jobs after a family member developed dementia, and 51% reduced their working hours. This was because they could not maintain economic activities due to their responsibilities of caring for dementia patients. Quitting a job or reduced working hours led to decreased income levels.

In terms of residential area, the proportion of those living in ‘*eup*’/‘*myeon*’ was significantly higher, which is consistent with a study [18] reporting that a large number of dementia sufferers and family members of home-dwelling dementia patients live in rural areas.

This study investigated the positive rate of depressive symptoms using the PHQ-9. The prevalence of depressive symptoms in the 2016 Korea National Health and Nutrition Examination Survey was 5.6%, which was measured by the PHQ-9 [19], while the prevalence of depressive symptoms in the 2017 KCHS was underestimated at 3.2%. Although these surveys used the same survey scale, the Korea National Health and Nutrition Examination Survey was conducted through one-to-one interviews in independent spaces, whereas the KCHS was conducted through one-to-one interviews in open spaces (possibly where family members were in the vicinity). Such an underestimation is thus thought to be due to the survey method.

However, it is difficult to assume that this was measured differentially according to whether or not families had to care for dementia patients, and thus it is assumed to be a bias toward the null.

In addition, the prevalence of depressive symptoms measured by the PHQ-9 and the rate of experience of depression measured by a single question were working in the same direction. An underestimation is expected because the survey was conducted in open spaces. However, in view of the fact that differential measurement errors did not occur depending on caring for a dementia patient, but non-differential errors occurred, the effects might be directed toward the null. Therefore, the results of this study are considered to be justified.

The positive rate of PHQ-9-measured depressive symptoms was higher in the middle-aged family members of home-dwelling dementia patients (4.4%) than in the middle-aged family members in the control group (1.9%). A similar previous study using the KCHS data found that the prevalence of depression diagnosed by physicians who did not use a scale was higher in the family members of home-dwelling dementia patients (5.0%) compared to the control group (2.5%) [20]. In addition, a study using the data from the 2012 and 2013 National Health and Wellness Survey, an online survey, in Japan with a PHQ-9 cut-off score of 10 points found that the positive rate of depressive symptoms was higher in the family members of home-dwelling dementia patients (14.2%) compared to the non-caregivers in the control group (8.6%) [21]. It is not possible to make an absolute comparison between the aforementioned previous study and this study due to the differences in tools and study methods. Nevertheless, similarities were observed between the two studies in that the prevalence of depressive symptoms was higher in the family members of home-dwelling dementia patients, and the positive rate of depressive symptoms was also higher in the family members of home-dwelling dementia patients despite the differences in medical systems and culture between the countries.

The ORs for depressive symptoms in the female family members of home-dwelling dementia patients and the control family members show that the positive rate of depressive symptoms was 2.3 times higher after adjusting for demographic variables of monthly drinking, current smoking, and adjustment variables. Daughters-in-law (sons and their wives) often cared for home-dwelling dementia patients, which can be interpreted as similar to the results of a study reporting that females had a high prevalence of depressive disorders in caring for dementia patients [4,22].

The PHQ-9 reflects all the nine criteria for the diagnosis of major depression according to the Diagnostic and Statistical Manual of Mental Disorders, 5th edition. An investigation into the characteristics of the severe depressive symptoms for each PHQ-9 question found that the positive rate on the questions, ‘Little or no interest or pleasure in doing things’, ‘Feeling tiered or having little energy’, and ‘Poor appetite or overeating,’ were higher in the family members of home-dwelling dementia patients. This is supported by the results of previous studies reporting that an increase in care-giving time is experienced as the most stressful among all the difficulties experienced by caregivers, and that the family caregivers of patients with dementia suffered from depression and loss of appetite [17,23].

The positive rate for the question, ‘Trouble falling or staying asleep, or sleeping too much’ was higher in the family members of home-dwelling dementia patients (8.3%), compared to the control group (3.6%). This result indicates that the family members of home-dwelling dementia patients tended to either lose sleep or oversleep. Sleep has commonly been reported as a risk factor for depression [24], and it is explained that dementia patients’ being awake at night is a common cause of sleep disorders in caregivers [9].

Question 8 in the PHQ-9 indicates psychomotor retardation, which is known to be biologically correlated with abnormalities in basal ganglia and dopaminergic pathways [25]. This is assessed that depression might be caused by caring for home-dwelling elderly with dementia, and biological abnormalities might not increase.

The positive rate for the question, ‘I would rather be better off dead or hurting myself’, which was related to suicide, was higher in the family members of home-dwelling dementia patients (1.5%) than in the control group (0.2%). A study by O’Dwyer et al. [10] reported that 16% of the family caregivers in the United States and Australia contemplated suicide more than once, and a fourth of those who contemplated suicide were likely to attempt suicide. The present findings are consistent with these results in that the prevalence of suicidal ideation was higher in the family members of home-dwelling dementia patients than in the control group.

Nevertheless, this study has several limitations. First, the presence or absence of home-dwelling dementia patients was based on only whether or not dementia patients diagnosed by a doctor were living at home, and the types and severity of dementia symptoms such as cognitive and mental behavior problems, and the degree of deterioration in physical function due to internal and surgical complications associated with dementia could not be identified. In addition, psychosocial factors such as dementia patients’ personality and their relationships with family members could not be considered.

Second, because there were no survey data regarding caring for home-dwelling dementia patients, such as what roles the middle-aged family members played as main caregivers, and whether or not any external caregiver visited the home and cared for the homed-welling dementia patient, it was not possible to identify the main caregivers. However, in view of the fact that the middle-aged family members, as the children generation of dementia patients, were living with dementia patients and caring for them, depression-related burden could be found in them.

Third, in order to enhance the comparison between the family members of home-dwelling dementia patients and the control group, adjustments were made for demographic variables, income level and economic activity variables, which are known as factors for burden in the family members of home-dwelling dementia patients, and monthly drinking and current smoking with p-value ≤ 0.25, found as the confounding variables in the univariate analysis. However, there were no survey data related to dementia patients, such as whether dementia patients were living in facilities, and whether caregivers were living at home, which are considered to be confounding variables. Therefore, adjustments could not be made for such variables, and they could not be compared.

Fourth, the 2017 KCHS is a cross-sectional study, and cannot clearly explain the causal relationship between depression and the presence or absence of home-dwelling dementia patients.

This study has representativeness as it used the data from the KCHS conducted in 254 areas across the country, and determined the positive rate of depressive symptoms in the middle-aged (40-59 years old) family members, who are the children of home-dwelling dementia patients. In particular, the results of this study confirmed that the positive rate of depressive symptoms was higher in the female family members of home-dwelling dementia patients, which is consistent with the results of previous studies.

Moreover, this study found that the response rate of severe depressive symptoms in most PHQ-9 questions was higher in the family members of home-dwelling dementia patients, indicating that the depressive symptoms in the family members of home-dwelling dementia patients were more serious compared to the control group. Therefore, preventive intervention approaches to the caregiver burden borne by the family members of home-dwelling dementia patients are needed in the future, and active interventions are needed to reduce depressive symptoms in the family members of home-dwelling dementia patients.

## Figures and Tables

**Table 1.** General characteristics of the subjects

Characteristics		Family with dementia (n=760)	Control family (n=76,516)	p-value
Age, mean±SE (yr)		51.0±0.3	49.5±0.0	<0.001
	40-49	260 (39.8)	36,442 (51.3)	<0.001
	50-59	500 (60.2)	40,074 (48.7)	
Gender	Men	371 (53.4)	34,892 (50.0)	0.176
	Women	389 (46.6)	41,624 (50.0)	
Household monthly income (10,000 Korean won)	<100	94 (11.0)	3,538 (2.9)	<0.001
	100-299	300 (37.2)	21,558 (22.6)	
	≥300	356 (51.9)	50,646 (74.5)	
Employment	Yes	591 (74.7)	61,284 (79.5)	0.014
	No	168 (25.3)	15,142 (20.5)	
Education level	≤ Elementary	92 (6.1)	5,047 (3.9)	<0.001
	Middle/high school	440 (57.3)	42,838 (51.2)	
	≥ College	227 (36.6)	28,537 (44.8)	
Household by generation	1	39 (3.9)	18,919 (17.4)	<0.001
	2	397 (50.9)	51,513 (75.2)	
	3	324 (45.2)	6,084 (7.4)	
Location	Urban	360 (72.7)	47,087 (83.0)	<0.001
	Rural	400 (27.3)	29,429 (17.0)	
Current smoker	Yes	176 (26.9)	16,096 (22.4)	0.027
	No	584 (73.1)	60,418 (77.6)	
Monthly alcohol drinking	Yes	434 (58.0)	46,373 (63.7)	0.016
	No	326 (42.0)	30,137 (36.3)	
Vigorous or moderate physical activity	Yes	545 (76.1)	57,393 (76.5)	0.837
	No	212 (23.9)	19,096 (23.5)	
Body mass index (kg/m^2^)	< 25	507 (68.1)	53,169 (70.5)	0.256
	≥25	244 (31.9)	22,546 (29.5)	

Values are presented as number (%).

**Table 2.** Depression question characteristics of the subjects

Variables	Categories	Family with dementia (n=760)	Control family (n=76,516)	p-value
PHQ-9	≥10	35 (4.4)	1,431 (1.9)	<0.001
	<10	725 (95.6)	75,085 (98.1)	
Experience of depressed mood^1^	Yes	90 (12.2)	4,084 (5.6)	
	No	669 (87.8)	72,418 (94.4)	<0.001

Values are presented as number (%). PHQ-9, Patient Health Questionnaire-9.

1 Experience of depressed mood were assessed by the following question: “In the last year, have you felt sadness or desperation for more than two weeks?”; Participants who answered “yes” were considered to have experience of depressed mood.

**Table 3.** OR for PHQ-9 detected depressive symptom between family with dementia patients at home and control family

	Depressive symptom^1^			
	Family with dementia, n (%)	Control family, n (%)	OR (95% CI)	aOR (95% CI)
Total middle-aged family^2^	35 (4.4)	1,431 (1.9)	2.46 (1.56, 3.88)	1.72 (1.03, 2.85)
Male family^3^	13 (3.2)	491 (1.5)	2.18 (0.99, 4.82)	1.08 (0.43, 2.70)
Female family^3^	22 (5.9)	940 (2.2)	2.75 (1.61, 4.68)	2.26 (1.26, 4.05)

OR, odds ratio; PHQ-9, Patient Health Questionnaire-9; CI, confidence interval; aOR, adjusted odds ratio.

1 Depressive symptom was evaluated by PHQ-9; a cut-off score ≥10 is positive.

2 Adjusted for age, gender, household monthly income, employment, education, household by generation, town, current smoking, and monthly alcohol drinking.

3 Adjusted for age, household monthly income, employment, education, household by generation, town, current smoking, and monthly alcohol drinking.

**Table 4.** Positive rate of Patient Health Questionnaire-9 (PHQ-9) each questions among middle-aged people between family with dementia patients at home and control family

Variables	Family with dementia (n=760)	Control family (n= 76,516)	p-value^1^
Little interest or pleasure in doing things	28 (3.7)	1,445 (2.0)	0.009
Feeling down, depressed, or hopeless	23 (2.6)	727 (0.9)	<0.001
Trouble falling or staying asleep, or sleeping too much	58 (8.3)	2,922 (3.6)	<0.001
Feeling tired or having little energy	47 (7.2)	3,017 (4.0)	0.002
Poor appetite or overeating	18 (2.1)	805 (1.1)	0.015
Feeling bad about yourself or that you are a failure or have let yourself or you family down	12 (0.9)	380 (0.5)	0.064
Trouble concentrating on things, such as reading the newspaper or watching television	9 (1.2)	293 (0.4)	0.001
Moving or speaking so slowly that other people could have noticed; Or the opposite being so fidgety or restless that you have been moving around a lot of more than usual	3 (0.2)	167 (0.2)	0.905
Thoughts that you would be better off dead, or of hurting yourself	12 (1.5)	194 (0.2)	<0.001

1 Chi-square test for PHQ-9 each question item.

## SUPPLEMENTARY MATERIALS

Korean version is available at http://www.e-epih.org/.
