# Supplementary material for: Characteristics of depressive symptoms in middle-aged family members of dementia patients: 2017 Korea Community Health Survey
Source: Epidemiol Health. 2020 May 15;42:e2020031. doi: 10.4178/epih.e2020031 (PMC7340617; doi:10.4178/epih.e2020031)
Supplement: Supplementary file 1 [file epih-42-e2020031-suppl1.pdf]

Volume: 42, Article ID: e2020031
https://doi.org/10.4178/epih.e2020031

Supplementary Material 1. Odds ratio for PHQ-9 detected depressive symptom between family with dementia patients at home and control family (age 30-69)

Depressive symptom				
Age	Family with dementia	Control family	Crude OR	Adjusted OR[1]
30-69	65 (5.4)	2,964 (2.2)	2.560 (1.795-3.650)	1.813 (1.246-2.636)

[1]Adjusted for age, gender, household monthly income, employment, education, household by generation, town, current smoking and monthly alcohol drinking

Supplementary Material 2. Odds ratio for PHQ-9 detected depressive symptom between family with dementia patients at home and control family (age 40-69)

Depressive symptom				
Age	Family with dementia	Control family	Crude OR	Adjusted OR[1]
40-69	61 (5.8)	2,273 (2.0)	2.921 (2.022-4.220)	1.925 (1.303-2.845)

[1]Adjusted for age, gender, household monthly income, employment, education, household by generation, town, current smoking and monthly alcohol drinking
