# Supplementary material for: Characteristics of depressive symptoms in middle-aged family members of dementia patients: 2017 Korea Community Health Survey
Source: Epidemiol Health. 2020 May 15;42:e2020031. doi: 10.4178/epih.e2020031 (PMC7340617; doi:10.4178/epih.e2020031)
Supplement: Supplementary file 2 [file epih-42-e2020031-suppl2.pdf]

Volume: 42, Article ID: e2020031
https://doi.org/10.4178/epih.e2020031

Characteristics of depressive symptoms in middle-aged family members of dementia patients: 2017 Korea Community Health Survey

재가 치매가족에서 중년층의 우울증상 특성 : 2017년 지역사회건강조사를 이용하여

박진범, 이원철, 정현숙, 홍나영, 배보영, 임현우*

가톨릭대학교 의과대학 예방의학교실

Corresponding Author's information

*For correspondence.

Address : Department of Preventive Medicine, College of Medicine, The Catholic University of Korea, 222 Banpo-daero, Seocho-gu, Seoul 06591, Korea

E-mail : y1693@catholic.ac.kr

Jinbeom Park: http://orcid.org/0000-0002-6928-2451

Won-Chul Lee: http://orcid.org/0000-0001-8052-2420

Hyunsuk Jeong: http://orcid.org/0000-0001-5274-3816

Nayoung Hong: http://orcid.org/0000-0001-6406-0461

Boyoung Bae: http://orcid.org/0000-0002-7068-7405

Hyeon Woo Yim: http://orcid.org/0000-0002-3646-8161

Volume: 42, Article ID: e2020031
https://doi.org/10.4178/epih.e2020031

초록

목적 : 재가 치매가족의 우울증상 특성은 명확하게 보고되지 않았다. 재가 치매 환자와 동거하는 중년층이 겪고 있는 우울증상 양성율과 PHQ-9 문항별 우울증상 특성을 알아보고자 하였다.

방법 : 2017 년 전국에서 수행된 지역사회건강조사 자료를 이용하였다. 조사 참여자 228,381 명에서 1 인가구를 제외한 40-50 대 77,433 명을 선택하고, 치매환자가구거주 질문에 무응답한 3 명, PHQ-9 문항에 무응답한 154 명을 제외한 후 77,276 명을 대상으로 하였다. 연구대상자는 재가 치매가족 760 명, 대조군 가족 76,516 명으로 구성되어 있다.

결과 : PHQ-9 으로 측정된 우울증상 양성율는 재가 치매가족에서 유의하게 더 높았다. (재가 치매가족: 4.4%, 대조군 가족 : 1.9%) 마지막으로, 연령, 성별, 가구소득, 경제활동, 학력수준, 세대유형, 거주지역, 현재흡연자, 월간음주를 보정하여 PHQ-9 로 측정한 우울증상은 대조군에 비해 재가 치매가족에서 1.72 배(95% CI 1.03-2.85) 유의하게 높았다. 재가 치매가족 중년여성은 대조군에 비해 우울증상 양성율이 2.26 배(95% CI 1.26-4.05)로 더 높았다. 또한, PHQ-9 문항에서 거의 매일 증상이 일어난다고 응답한 대상자는 자기비난, 좌불안석 또는 처진느낌 문항을 제외하고 흥미상실, 우울감, 수면문제, 피로감, 식욕문제, 자살생각 문항은 대조군에 비해 유의하게 더 높았다.

결론 : 재가 치매가족의 우울증 완화를 위한 좀더 적극적인 개입이 필요하다.

주제어: 재가 치매가족, 우울증상 양성율, PHQ-9, 지역사회건강조사

Keywords : family with dementia patient, depressive symptom, PHQ-9, CHS

Volume: 42, Article ID: e2020031
https://doi.org/10.4178/epih.e2020031

서론

치매는 전 세계적으로 4,680 만 명이 넘는 사람들에게 영향을 주는 치명적인 질병이다. 이 수치는 20 년마다 2 배로 증가하여 2030 년에는 7500 만명, 2050 년에는 약 1 억 3 천만명에 도달 할 것으로 예상된다 [1]. 국내 현황을 살펴보면 2017 년 우리나라에서 치매환자로 추정되는 환자수는 전국 노인인구 7,066,201 명중 702,436 명이며, 치매환자가족은 배우자, 자녀, 손주를 포함하여 약 350 만명에 이르는 것으로 보고되었다 [2].

한국에서는 치매에 대한 정책으로 2017 년 치매 국가책임제 추진계획을 실행하였고 치매환자와 치매환자 가족에 대한 다양한 서비스가 제공되기 시작했다. 치매환자가족을 위한 자조모임, 휴가제 등 여러가지 프로그램을 시행 중이지만 부담을 완화하는 효과가 미비한 실정이다 [3].

재가 치매 가족에서 아들 내외(주로 며느리)가 부양하는 경우가 많고 동거 가족의 50-60%가 40-50 대 중년층으로 실질적 경제활동을 하는 사람들이 치매 환자 부양부담을 지고있다 [4]. 그로 인해 경제활동의 제한과 치매진료비로 인한 경제적 부담을 경험한다. [5]

치매환자가족은 치매 환자 관리에 중심적인 역할을 하며 환자의 인지 기능 손상이 악화됨에 따라 지속적인 병간호에 필요한 신체적, 정신적 노력이 증가하여 스트레스와 우울증의 위험이 증가하는 것으로 나타났으며 [6], 치매노인을 돌보는 경우가 만성질환, 뇌졸중이 있는 노인을 돌보는 경우보다 부양 부담이 더 높은 것으로 보고되었다 [7].

우울증상은 치매노인 부양자가 경험하는 가장 일반적인 정신과적 증상으로 치매노인 가족의 75%가 이를 경험하고 있다 [8]. 그 외에 치매가족 보호자의 약 60 %가 수면 장애가 있었으며, [9] 16%는 한 번 이상 자살생각을 하였다 [10]. 또한 건강 상태가 좋지 않았고 [5, 11] 뇌졸증 위험이 높았다 [12]. 이처럼 치매환자 가족은 신체적 부담 뿐만 아니라 우울증을 포함한 여러가지 정신적인 부담이 보고되었지만, 우울증상 특성은 명확하게 보고되지 않았다. 이에 본 연구는 우리나라를 대표할 수 있는 2017 년 지역사회 건강조사 자료를 이용하여 재가 치매가족의 PHQ-9 으로 측정한 우울증상 양성율과 PHQ-9 문항별 특성을 비교하여 우울증 특성을 파악하고자 한다.

Volume: 42, Article ID: e2020031
https://doi.org/10.4178/epih.e2020031

재료 및 방법

1. 연구설계

본 연구는 2017 년 전국에서 수행된 지역사회건강조사 자료를 이용하여 재가 치매가족 중년층의 우울증과 PHQ-9 문항별 특성을 확인하기 위한 단면연구이다.

2. 연구 대상 및 자료 수집

지역사회건강조사는 지역보건법에 의거하여 2008 년부터 매년 시행되고 있는 조사로 목표 모집단은 당 해 연도 7 월을 기준으로 만 19 세이상의 성인이다. 각 보건소별로 동·읍·면과 주택유형으로 층화하여 표본지점을 할당하고 추출된 표본지점에서 한 지점당 평균 5 가구를 표본으로 선정한후, 선정된 19 세 이상 모든 가구원을 조사하여 약 900 명을 조사하였다. [13] 연구에서는 2017 년 지역사회건강조사에 참여한 만 19 세 이상 성인 남, 여를 대상으로 하였으며, 치매환자 부양 가능성이 없는 1 인 가구는 제외하고 20 대, 30 대는 부양자가 아닐 가능성이 있으며, 치매환자가 65 세 이상 노인인구에서 분포하고 있다는 점을 고려하여 치매 환자 자녀세대인 중년층(40 대, 50 대)을 대상으로 하였다. 따라서 조사 참여자 228,381 명 중 1 인 가구를 제외하고 40-50 대를 우선 추출한 후 치매환자가구거주 질문에 무응답한 3 명, PHQ-9 문항에 무응답한 157 명을 제외한 총 77,276 명 전수를 대상으로 하였다. 현재 의사로부터 진단받은 치매환자와 함께 거주하는 가족을 재가 치매가족으로 치매 환자가 함께 거주하고 있지 않은 가족을 대조군 가족으로 정의하였으며, 재가 치매가족 760 명, 대조군 가족 76,516 명으로 구성되어 있다. (Figure.1)

Volume: 42, Article ID: e2020031
https://doi.org/10.4178/epih.e2020031

3. 연구도구

1) 일반적 특성

일반적 특성으로 성별은 남자, 여자로 분류하였다. 연령은 40-49 세는 40 대, 50-59 세는 50 대로 구분하였다. 월 평균 가구소득은 100 만원 미만, 100 만원 이상-300 만원 미만, 300 만원 이상으로 구분하였다. 경제활동은 직업이 있을 경우 '경제활동 하고 있음'으로 학생/재수생, 주부, 무직으로 응답한 경우 '경제활동 하지 않음'으로 구분하였다. 교육수준은 초등학교 이하, 중/고등학교, 대학교 졸업 이상으로 분류하였다. 세대유형은 1 인 가구를 제외하고 '응답자+미혼형제자매', '부부(응답자+배우자)', '부부+기타 친인척', ' 부부+미혼 형제자매', '응답자+기타 친인척', '기타 : 그외 1 세대 가구'를 1 세대 가구, '부부+미혼자녀', '부부+양친', '조부모+미혼 손자녀', '편부+미혼자녀', '부부+편부/편모', '편조부/편조모+미혼 손자녀', '편모+미혼자녀', '부부+자녀+부부의 형제 또는 자매', '기타 : 그외 2 세대 가구'를 2 세대 가구, '부부+미혼자녀+양친', '부부+미혼자녀+편부/편모', '기타 : 그외 3 세대 가구'를 3 세대 가구로 정의 하였다. 거주지 유형은 동, 읍/면으로 분류하였다. 현재흡연자는 "현재 담배를 피웁니까?" 문항에 '매일피움'과 '가끔 피움'으로 응답한 사람을 현재흡연자로 정의하였다. 월간음주는 최근 1 년동안 한달에 1 회 이상 술을 마신 적이 있는 사람을 음주를 한 것으로 정의하였다. 중등도 이상 신체활동 실천 여부는 최근 1 주일 동안 중등도 신체활동을 1 일 총 30 분 이상, 주 5 일 이상 실천하거나 또는 격렬한 신체활동을 1 일 총 20 분 이상, 주 3 일 이상 실천한 것을 중등도 이상 신체활동을 하는 것으로 하였다. 체질량지수는 대상자의 신장과 체중을 설문조사로 수집한 체중(㎏)을 신장(m)의 제곱으로 나누어 계산하여 체질량지수가 25(kg/m2) 이상은 비만, 25(kg/m2)미만은 정상으로 구분하였다.

2) 우울증상

우울증상은 우울증 진단점수 PHQ-9(Patient Health Questionnaire-9)을 이용하여 측정한 값을 분석하였다. PHQ-9 은 주요우울장애의 선별 도구로 널리 쓰이고 있으며 민감도(88%)와 특이도(88%)가 높은 것으로 알려져 있다 [14]. 9 가지 문항으로 구성되어 있다. 각 항목의 점수는 0에서 3까지이며, 총 0에서 27점 사이의 점수가 산출된다. 우울증의 기준은 가벼운 우울증 5-9 점, 중간정도 우울증 10-19 점, 심한 우울증 20-27 점으로 본 연구에서는 주요 우울 장애(MDD) 기준인 10 점 이상을 절단점으로 하였다. 우울증상 특성을 파악하기 위해 PHQ-9 각 문항별로 0~2점은 음성, 3점은 양성으로 재분류 하여 응답 비율을 파악하였다. 본 연구에서의 Cronbach’s alpha=0.78 이다.

우울감 경험은 최근 1 년 동안 연속 2 주 이상 일상생활에 지장이 있을 정도로 슬프거나 절망감 등을 느낀 적이 있는 사람을 우울감 경험이 있다고 정의하였다.

4. 통계분석방법

본 연구의 통계분석은 SAS (SAS Institute Inc, Cary, Nc, USA) version 9.4 를 사용하여 가중치를 고려한 복합표본설계(Complex Sample Design) 방법으로 분석하였으며, 통계적 유의수준은 $P < 0.05$ 이다.

치매환자 가구거주 유무에 따른 두 그룹 간의 특성을 비교하기 위해 빈도와 백분율을 제시하였고, 연속형 변수는 평균과 표준오차를 제시하였다.

두 그룹간의 차이 비교는 범주형 변수는 카이제곱 검정(Rao-Scott chi-square test)을, 연속형 변수는 t-검정(t-test)을 실시하였다.

PHQ-9 으로 측정한 우울증상 유병율과 문항별 양성율 차이는 카이제곱 검정(Rao-Sc chi-square test)을 실시하였다.

혼란변수는 인구사회학적 변수와 재가 치매가족에서 부담 요소 [15]로 알려진 소득수준과 경제활동 변수 그리고 단변량 분석에서 p 값 0.25 이하인 월간음주, 현재흡연자를 보정변수로 하였으며, 치매환자가구거주 유무에 따른 우울증상의 연관성을 확인하기 위해 다변수 로지스틱 회귀분석(Multivariable logistic regression)을 실시하였다.

Volume: 42, Article ID: e2020031
https://doi.org/10.4178/epih.e2020031

본 연구는 ‘가톨릭대학교 임상연구심사위원회’의 승인을 받았다. (승인번호: MC19ZESI0051)

결과

1. 재가 치매가족과 대조군 가족의 일반적 특성

두 집단에서 일반적 특성에 차이가 있는지 분석한 결과 연령, 가구소득, 경제활동, 학력수준, 세대유형, 거주지역, 현재흡연자, 월간음주, 진단된 질병의 개수 변수에서 유의한 차이가 있었다(p=0.05). 연령대별로는 재가 치매가족 50~59세 60.2%, 40~49세 39.8% 순이었으나, 대조군 가족은 40~49세 51.3%, 50~59세 48.7% 순이었다. 가구소득 100만원 미만 구성비는 재가 치매가족 11.0%, 대조군 가족 2.9%로 재가 치매가족에서 높았으며, 300만원 이상 구성비는 재가 치매가족 51.9%, 대조군 가족 74.5%로 대조군 가족에서 높았다. 경제활동을 하지 않는 대상자의 구성비는 재가 치매가족 25.3%, 대조군 가족 20.5%로 재가 치매가족에서 높았다. 현재흡연자는 재가 치매가족 26.9%, 대조군 가족 22.4%로 재가 치매가족에서 높았다. 월간음주을 하는 대상자의 구성비는 재가 치매가족에 58.0%, 대조군 가족 63.7%로 대조군 가족에서 높았다. (Table.1)

2. 우울증 문항 특성

단일 문항으로 측정한 우울감 경험율은 재가 치매가족 12.2%, 대조군 가족 5.6%로 재가 치매가족에서 높았다. (Table.2) PHQ-9으로 측정한 우울증상 유병율은 재가 치매가족 4.4%, 대조군 가족 1.9%로 재가 치매가족에서 높았다. 성별로는 남성에서 재가 치매가족 3.2%, 대조군 가족 1.5%로 재가 치매가족에서 높았으며, 여성에서는 재가 치매가족 5.9%, 대조군 가족 2.2%로 재가 치매가족 여성에서 높았다.

치매환자가구거주 유무에 따른 우울증상 유병율을 알아보기 위해 연령, 성별, 가구소득, 경제활동, 학력수준, 세대유형, 거주지역, 현재흡연자, 월간음주, 진단된 질병의 개수 변수를 혼란변수로 하여 다변수 로지스틱 회귀분석을 실시하였다.

재가 치매가족에서 대조군 가족보다 PHQ-9으로 측정한 우울증상 유병율이 교차비 2.457(95% CI 1.557-3.878), 보정 후 1.716(95% CI 1.032-2.853)로 유의하게 높았다. 40대와 50대로 제한했던 선정기준에 대한 민감도 분석을 시행하였다. 연령을 30-69세, 40에서 69세로 바꾸어도 유사한 결과를 보였다(Supplementary Materials 1 and 2).

보정 전 대조군 가족 여성에 비해 재가 치매가족 여성에서 교차비 2.75(95% CI 1.61-4.67),

보정 후 교차비 2.264(95% CI 1.265-4.053)로 유의하게 높았다. 남성에서는 유의하지 않았다. (Table.3)

3. 재가 치매가족과 대조군 가족의 PHQ-9 문항별 특성

두 집단에서 문항별로 차이가 있는지 분석한 결과 흥미상실, 우울감, 수면문제, 피로감, 식욕문제는 재가 치매가족에서 높았다. 더군다나 거의 매일 죽음에 대해 생각하는 비율도 재가 치매가족 1.5%, 대조군 가족 0.2%로 재가 치매가족에서 높았다. '내 자신이 나쁜 사람이라는 느낌 혹은 내 자신을 실패자라고 느끼거나, 나 때문에 나 자신이랑 내 가족이 불행하게 되었다는 느낌' (p=0.064), '남들이 알아챌 정도로 거동이나 말이 느림 또는 반대로 너무 초조하고 안절부절하지 못해서 평소보다 많이 돌아다니고 서성거림' (p=0.905)문항을 제외한 모든 문항에서 유의한 차이가 있었다. (Table 4/Figure 2)

고찰

본 연구는 지역사회건강조사 2017년 자료를 활용하여 중년층에서 재가 치매가족과 대조군 가족의 PHQ-9으로 측정한 우울증상 양성율의 차이를 알아보고, 재가 치매가족의 우울증상 특성을 파악하기 위해 PHQ-9 문항별 양성율을 확인한 연구이다.

본 연구에서 재가 치매가족 중년층의 평균 연령은 51.0세로 주부양자 가족을 연구 대상으로 한 Kim et al. [16] 의 연구의 치매 주부양자 평균 연령 52.5세와 차이가 없었으며, 주보호자는 대부분 중년층에 위치하여 치매환자를 돌보는 것을 확인할 수 있었다.

가구소득 및 경제활동은 재가 치매가족에서 유의하게 낮았다. Choi et al. [17]의 연구에서 치매 발병 후 보호자의 27%가 퇴사하고, 51%가 노동시간을 축소한다고 보고하였는데 이는 치매 환자 간병으로 경제활동을 유지할 수 없기 때문에 퇴사 또는 노동시간 축소하여 소득수준이 감소하였음을 확인할 수 있었다.

거주지역은 재가 치매가족이 읍/면에서 유의하게 높았는데, 치매 유병자와 재가 치매가족의 많은 수가 농촌에 거주한다는 보고한 연구와 일치한다 [18].

본 연구는 PHQ-9으로 우울증상 양성율을 확인하였는데, 2016년 국민건강영양조사에서 PHQ-9으로 조사한 우울증상 유병률은 5.6%이며 [19], 2017년 지역사회건강조사에서는 3.2%로 과소추정 되고 있다. 이는 조사도구는 같은데 국민건강영양조사는 독립된 공간에서 1대1 면접으로 측정된 반면, 지역사회 조사는 개방된 공간(가구 구성원이 주변에 있을 가능성이 있음)에서 1대1 면접으로 조사 방식에 따른 과소추정으로 사료된다. 그러나 치매부양 여부에 따라 차별적으로 측정되었다고 보기 어려워 null을 향해 작용하는 바이어스로 추정된다. 또한 PHQ-9으로 측정된 우울증상 유병률과 단일문항으로 측정한 우울감 경험율 값이 똑같은 방향으로 작동되고 있는데, 이는 개방된 공간(가구 구성원이 주변에 있을 가능성이 있음)에서 조사를 진행하여 과소추정이 예상되지만 치매부양여부에 따라 차별적 측정편의가 발생하지 않고 비차별적으로 발생하기 때문에 효과는 null을 향해 작용 할 것이기 때문에 본 연구 결과는 당위성이 있다고 판단된다.

PHQ-9으로 측정한 우울증상 양성율은 재가 치매가족 중년층에서 4.4%, 대조군 가족 중년층에서 1.9%로 재가 치매가족 중년층에서 우울증상 양성율이 더 높았다. 지역사회건강조사 데이터를 사용한 유사한 국내 선행연구에서 척도를 이용하지 않은 의사로부터 진단받은 우울증은 재가 치매가족 5.0%, 대조군 가족 2.5%로 재가 치매가족에서 더 높았다 [20]. 또한 온라인 설문조사인 일본 2012~2013년 국민건강조사(National Health and Wellness Survey)

자료를 이용하여 PHQ-9 절단 점수를 10점으로 한 연구에서 재가 치매가족 14.2%, 대조군 가족 8.6%로 재가 치매가족에서 우울증상 양성율의 비율이 더 높았다 [21]. 앞의 선행 연구와 본 연구는 도구와 조사 방법의 차이로 절대적인 비교는 불가능하다. 그럼에도 재가 치매가족에서 우울증상의 비율이 더 높다는 점에서 유사한 경향을 확인할 수 있었으며, 의료제도 및 문화적 차이에도 불구하고 재가 치매가족에서 우울증상 양성율이 높은 것을 확인할 수 있다.

재가 치매가족 여성과 대조군 가족 여성의 교차비를 확인한 결과 우울증상 양성율이 인구사회학적 변수와 월간음주, 현재흡연자, 진단된 보정변수를 보정 후 2.3배 높았다. 재가 치매가족에서는 며느리(아들 내외)가 부양하는 경우가 많으며, 여성이 치매 환자 부양에 있어서 우울장애가 높다고 보고한 연구와 유사한 결과로 해석할 수 있다. [4,22]

PHQ-9는 정신 장애의 진단 및 통계 매뉴얼 제 5 판 (DSM-5)의 주요 우울증에 대한 9 가지 기준을 반영한다. PHQ-9 문항별 심각한 우울증상 특성을 확인 한 결과, '일을 하는 것에 대한 흥미나 재미가 거의 없음', '피곤함, 기력이 저하됨', '식욕 저하 혹은 과식' 등의 문항은 재가 치매가족에서 높았는데, 보호자가 겪는 어려움 중 간병시간의 증가가 가장 부담스럽다는 조사결과와 간병을 하는 가족이 우울증, 식욕감퇴 등의 질병을 가지고 있다는 보고가 이를 뒷받침해 준다. [17,23]

'잠들기 어렵거나 자꾸 깨어남, 혹은 너무 많이 잠' 문항에서 재가 치매가족 8.3%, 일반가족 3.6%로 재가 치매가족에서 높았다. 이는 재가 치매가족에서 수면 부족 또는 과수면의 경향이 있음을 알 수 있다. 수면은 일반적으로 우울증의 위험인자로 보고되고 있으며 [24], 치매환자의 야간 각성이 간병인의 수면 장애를 유발하는 일반적인 원인으로 설명하고 있다 [9].

PHQ-9의 8번 문항은 정신운동지제 소견을 나타내는 것으로 생물학적으로 기저핵과 도파민 회로 이상과 상관 관계가 있는 것으로 알려져 있다 [25]. 이는 재가 치매노인 부양으로 인한 우울증상으로 생물학적 이상소견이 증가되지 않는 것으로 판단된다.

자살과 관련 있는 '나는 차라리 죽는 것이 낫겠다는 등의 생각 혹은 어떤 면에서건 당신스스로 에게 상처를 주는 생각들' 문항은 재가 치매가족 1.5%, 대조군 가족 0.2%로 재가 치매가족에서 높았다. O'Dwyer ST et al. [10] 의 연구에서 미국과 호주 가족간병인 중 16%는 한 번 이상 자살 생각을 했으며, 자살 생각을 한 사람 중 1/4은 자살 시도 가능성이 높은 것으로 보고되었다. 일관적으로 재가 치매가족에서 자살생각이 높다는 점에서 유사한 경향

Volume: 42, Article ID: e2020031
https://doi.org/10.4178/epih.e2020031
을 보였다.

본 연구의 제한점은 다음과 같다. 첫째, 치매환자 가구거주 유무는 의사로부터 진단받은 치매환자의 거주 유무만 확인하며 치매환자의 인지, 정신행동문제 등의 치매증상의 종류 및 심각도, 치매에 따른 내외과적 합병증 등으로 인한 신체기능 저하 정도를 알 수 없었다. 또한 치매환자의 성격, 평소 가족과의 관계 등의 심리사회적 요인도 고려할 수 없었다. 둘째, 중년층이 주부양자로서 어떤 역할을 하는지 외부에서 간병인이 방문하는지 등의 부양과 관련된 조사 자료가 없어서 주부양자를 확인할 수 없었다. 그러나 주부양자를 확인할 수 없지만, 같이 거주하며 치매환자를 부양한다는 점에서 자녀세대인 중년층에서 우울 관련 부담을 확인할 수 있었다. 셋째, 재가 치매가족과 대조군 가족의 비교성을 높이기 위해 인구사회학적 변수와 재가 치매가족에서 부담 요소로 알려진 소득수준과 경제활동 변수 그리고 단변량 분석에서 p값 0.25이하인 월간음주, 현재흡연자를 혼란변수로 보정하였다. 하지만 혼란변수로 판단되는 치매 환자 시설 거주 유무와 간병인 거주 유무 등의 치매 환자와 관련된 조사 자료가 없어 관련 변수를 보정하지 못하여 비교하지 못한 제한점이 있다. 넷째, 2017년 지역사회건강조사는 단면 연구를 통해 확인된 결과로 치매환자 가구거주 유무에 따른 우울증상의 인과 관계를 명확하게 설명할 수 없다.

본 연구는 전국 254개 지역에서 실시하는 지역사회건강조사를 이용하여 대표성을 가지고 있으며, 치매 환자 자녀세대 연령대인 중년층(40~59세)을 대상으로 재가 치매가족의 우울증상 양성율을 확인할 수 있었다. 특히 기존 연구에서 보고된 결과와 같은 방향으로 재가 치매가족 여성에서 우울증상 양성율이 더 높은 것을 확인할 수 있었다.

더군다나 재가 치매가족에서 대부분의 PHQ-9 문항에서 심각한 우울증상 응답이 더 높은 것을 확인할 수 있었으며, 이는 대조군 가족과 비교하여 재가 치매가족의 우울증상이 더 심각하다는 것을 의미한다. 따라서 향후 재가 치매가족의 부양부담에 대한 예방적 차원의 중재 접근이 필요하며, 재가 치매가족의 우울증상을 낮추기 위한 적극적인 개입이 필요할 것이다.

Volume: 42, Article ID: e2020031
https://doi.org/10.4178/epih.e2020031

참고문헌

1. Alzheimer's Association. 2018 Alzheimer's disease facts and figures. Alzheimers Dement 2018;14:367-429.

2. National Institute of Dementia. 2017 National institute of dementia annual report. Seoul: National Institute of Dementia; 2017 (Korean).

3. Lim KC. Influencing factors on care burden among family caregivers for elders with dementia: focusing on family caregivers using a support center for dementia. J Korean Acad Soc Nurs Educ 2019;25:136-147 (Korean).

4. Yoon B, Shim YS, Kim YD, Lee KO, Na SJ, Hong YJ, et al. Who takes care of patients with dementia in Korea: a study on the present state of patients with dementia living alone and primary caregivers. Dement Neurocogn Disord 2012;11:13-17 (Korean).

5. Park MH, Go YH, Jeong MR, Lee SJ, Kim SH, Kim J. Influencing factors and risk of caregiver burden of family caregivers for patient with dementia. Korean J Fam Welf 2017;22:431-448 (Korean).

6. Huang CY, Sousa VD, Perng SJ, Hwang MY, Tsai CC, Huang MH, et al. Stressors, social support, depressive symptoms and general health status of Taiwanese caregivers of persons with stroke or Alzheimer's disease. J Clin Nurs 2009;18:502-511.

7. Lee HS. Caregiver burden in caring for elders before and after long-term care service in Korea. J Korean Acad Nurs 2012;42:236-247 (Korean).

8. Lee SA, Kim HS. Effects of a dementia family education program for dementia recognition, burden, and depression in caregivers of elders with dementia. J Korean Acad Psychiatr Ment Health Nurs 2017;26:14-23 (Korean).

9. Gehrman P, Gooneratne NS, Brewster GS, Richards KC, Karlawish J. Impact of Alzheimer disease patients' sleep disturbances on their caregivers. Geriatr Nurs 2018;39:60-65.

10. O'Dwyer ST, Moyle W, Zimmer-Gembeck M, De Leo D. Suicidal ideation in family carers of people with dementia. Aging Ment Health 2016;20:222-230.

11. Lee HJ, Lee JW, Lee JY. Family caregiver's burden for the elderly with dementia: moderating effects of social support. Soc Sci Res Rev 2015;26:345-367 (Korean).

12. Hong I, Han A, Reistetter TA, Simpson AN. The risk of stroke in spouses of people living with dementia in Korea. Int J Stroke 2017; 12:851-857.

13. Kim YT, Choi BY, Lee KO, Kim H, Chun JH, Kim SY, et al. Overview of Korean Community Health Survey. J Korean Med Assoc 2012;55:74-83 (Korean).

14. Park SJ, Choi HR, Choi JH, Kim KW, Hong JP. Reliability and validity of the Korean version of the Patient Health Questionnaire-9 (PHQ-9). Anxiety Mood 2010;6:119-124 (Korean).

15. Lee SY, Park HR, Choi SE, Lee SJ. Depression and depression relating variables for caregivers of patients with dementia in home. J Korean Acad Psychiatr Ment Health Nurs 2005;14:250-259 (Korean).

16. Kim SY, Yoon HS, Kim JS. Factors affecting life satisfaction and depression of caregivers of the elderly with dementia: a comparative study of Korean and American caregivers. J Korea Gerontol Soc 2003;23:193-221 (Korean).

17. Choi H, Yang YS, Kim HJ, Na HR, Shim YS, Park KW, et al. The survey for current state and dognition of activities of daily living in dementia patients- “Il-sang-ye-chan” campaign. Dement Neurocogn Disord 2013;12:47-51 (Korean).

18. Kim J, Ryu W, Choi Y. The effect of caregiver burden on depression of dementia family caregivers: the moderating role of perceived public support. Korean J Gerontol Soc Welf 2018;73:171-191 (Korean).

19. Korea Centers for Disease Control and Prevention. Korea health statistics 2016: Korea National Health and Nutrition Examination Survey (KNHANES VII-1). Sejong: Korea Centers for Disease Control and Prevention; 2016, p. 106 (Korean).

20. Jang SI, Bae HC, Shin J, Jang SY, Hong S, Han KT, et al. Depression in the family of patients with dementia in Korea. Am J Alzheimers Dis Other Demen 2016;31:481-491.

21. Goren A, Montgomery W, Kahle-Wrobleski K, Nakamura T, Ueda K. Impact of caring for persons with Alzheimer’s disease or dementia on caregivers’ health outcomes: findings from a community based survey in Japan. BMC Geriatr 2016;16:122.

22. Lee AS, Kim HG. Care-givers’ attitude & determinants about the burden of the caring for senile dementia patients. Health Med Soc 2003;13:29-60 (Korean).

23. Alzheimer’s Disease International. World Alzheimer report 2009: the global prevalence of dementia [cited 2020 May 15]. Available from: https://www.alz.co.uk/research/world-report-2009.

24. Beaudreau SA, Spira AP, Gray HL, Depp CA, Long J, Rothkopf M, et al. The relationship between objectively measured sleep disturbance and dementia family caregiver distress and burden. J Geriatr Psychiatry Neurol 2008;21:159-165.

25. Buyukdura JS, McClintock SM, Croarkin PE. Psychomotor retardation in depression: biological underpinnings, measurement, and treatment. Prog Neuropsychopharmacol Biol Psychiatry 2011; 35:395-409.

Volume: 42, Article ID: e2020031
https://doi.org/10.4178/epih.e2020031

Table 1. General characteristics of the subjects

Variables	Categories	Family with dementia n = 760	Control family n = 76,516	*P*-value
		n (%) or M±SE	n (%) or M±SE	
Age, year		51.0±0.3	49.5±0.0	<0.001
Age group, year	40-49	260 (39.8)	36,442 (51.3)	<0.001
	50-59	500 (60.2)	40,074 (48.7)	
Gender	Male	371 (53.4)	34,892 (50.0)	0.176
	Female	389 (46.6)	41,624 (50.0)	
Household monthly income (10,000 won)	<100	94 (11.0)	3,538 (2.9)	<0.001
	100-299	300 (37.2)	21,558 (22.6)	
	≥300	356 (51.9)	50,646 (74.5)	
Employment	Yes	591 (74.7)	61,284 (79.5)	0.014
	No	168 (25.3)	15,142 (20.5)	
Education Level	≤Elementary	92 (6.1)	5,047 (3.9)	<0.001
	Middle/High school	440 (57.3)	42,838 (51.2)	
	≥College	227 (36.6)	28,537 (44.8)	
Household by generation	one generational household	39 (3.9)	18,919 (17.4)	<0.001
	two generational household	397 (50.9)	51,513 (75.2)	
	three generational household	324 (45.2)	6,084 (7.4)	
Location	Urban	360 (72.7)	47,087 (83.0)	<0.001
	Rural	400 (27.3)	29,429 (17.0)	
Current smoker	Yes	176 (26.9)	16,096 (22.4)	0.027
	No	584 (73.1)	60,418 (77.6)	
Monthly alcohol drinking	Yes	434 (58.0)	46,373 (63.7)	0.016
	No	326 (42.0)	30,137 (36.3)	
Vigorous or Moderate physical activity	Yes	545 (76.1)	57,393 (76.5)	0.837
	No	212 (23.9)	19,096 (23.5)	
Body mass index	< 25 kg/m2	507 (68.1)	53,169 (70.5)	0.256
	≥25 kg/m2	244 (31.9)	22,546 (29.5)	

† T-test for age
‡ Chi-square for gender, age group, household monthly income, employment, education level, household by generation, location, current smoker, monthly alcohol drinking, vigorous or moderate physical activity and body mass index
f

Volume: 42, Article ID: e2020031
https://doi.org/10.4178/epih.e2020031

Table 2. depression question characteristics of the subjects

Variables	Categories	Family with dementia n = 760	Control family n = 76,516	*P*-value
		n (%)	n (%)	
PHQ-9	≥10	35 (4.4)	1,431 (1.9)	<0.001
	＜10	725 (95.6)	75,085 (98.4)	
Experience of depressed mood	Yes	90 (12.2)	4,084 (5.6)	<0.001
	No	669 (87.8)	72,418 (94.4)	

* Depressive symptom was evaluated by Patient Health Questionnaire-9;a cut off score ≥10 is positive
Experience of depressed mood were assessed by the following question: “In the last year, have you felt sadness or desperation for more than two weeks?”. Participants who answered “yes” were considered to have Experience of depressed mood.

† Chi-square PHQ-9 and experience of depressed mood

Volume: 42, Article ID: e2020031
https://doi.org/10.4178/epih.e2020031

Table 3. Odds ratio for PHQ-9 detected depressive symptom between family with dementia patients at home and control family

	Depressive symptom			
	Family with dementia	Control family	Crude OR	Adjusted OR
Total middle-aged family‡	35 (4.4)	1,431 (1.9)	2.457 (1.557-3.878)	1.716 (1.032-2.853)
Male family§	13 (3.2)	491 (1.5)	2.180 (0.987-4.817)	1.080 (0.432-2.697)
Female family§	22 (5.9)	940 (2.2)	2.746 (1.612-4.678)	2.264 (1.265-4.053)

* Depressive symptom was evaluated by Patient Health Questionnaire-9;a cut off score ≥10 is positive

† Values are presented as number (%) or odds ratio (95% confidence interval).

‡ Adjusted for age, gender, household monthly income, employment, education, household by generation, town, current smoking and monthly alcohol drinking

§ Adjusted for age, household monthly income, employment, education, household by generation, town, current smoking and monthly alcohol drinking

Volume: 42, Article ID: e2020031
https://doi.org/10.4178/epih.e2020031

Table 4. Positive rate of PHQ-9 each questions among middle-aged people between family with dementia patients at home and control family

Variables	Family with dementia n = 760	Control family n= 76,516	*P*-value
1. Little interest or pleasure in doing things	28 (3.7)	1,445 (2.0)	0.009
2. Feeling down, depressed, or hopeless	23 (2.6)	727 (0.9)	<0.001
3. Trouble falling or staying asleep, or sleeping too much	58 (8.3)	2,922 (3.6)	<0.001
4. Feeling tired or having little energy	47 (7.2)	3,017 (4.0)	0.002
5. Poor appetite or overeating	18 (2.1)	805 (1.1)	0.015
6. Feeling bad about yourself or that you are a failure or have let yourself or you family down	12 (0.9)	380 (0.5)	0.064
7. Trouble concentrating on things, such as reading the newspaper or watching television	9 (1.2)	293 (0.4)	0.001
8. Moving or speaking so slowly that other people could have noticed. Or the opposite being so fidgety or restless that you have been moving around a lot of more than usual	3 (0.2)	167 (0.2)	0.905
9. Thoughts that you would be better off dead, or of hurting yourself	12 (1.5)	194 (0.2)	<0.001

† Chi-square test for PHQ-9 each question item

Volume: 42, Article ID: e2020031
https://doi.org/10.4178/epih.e2020031

Figure legends

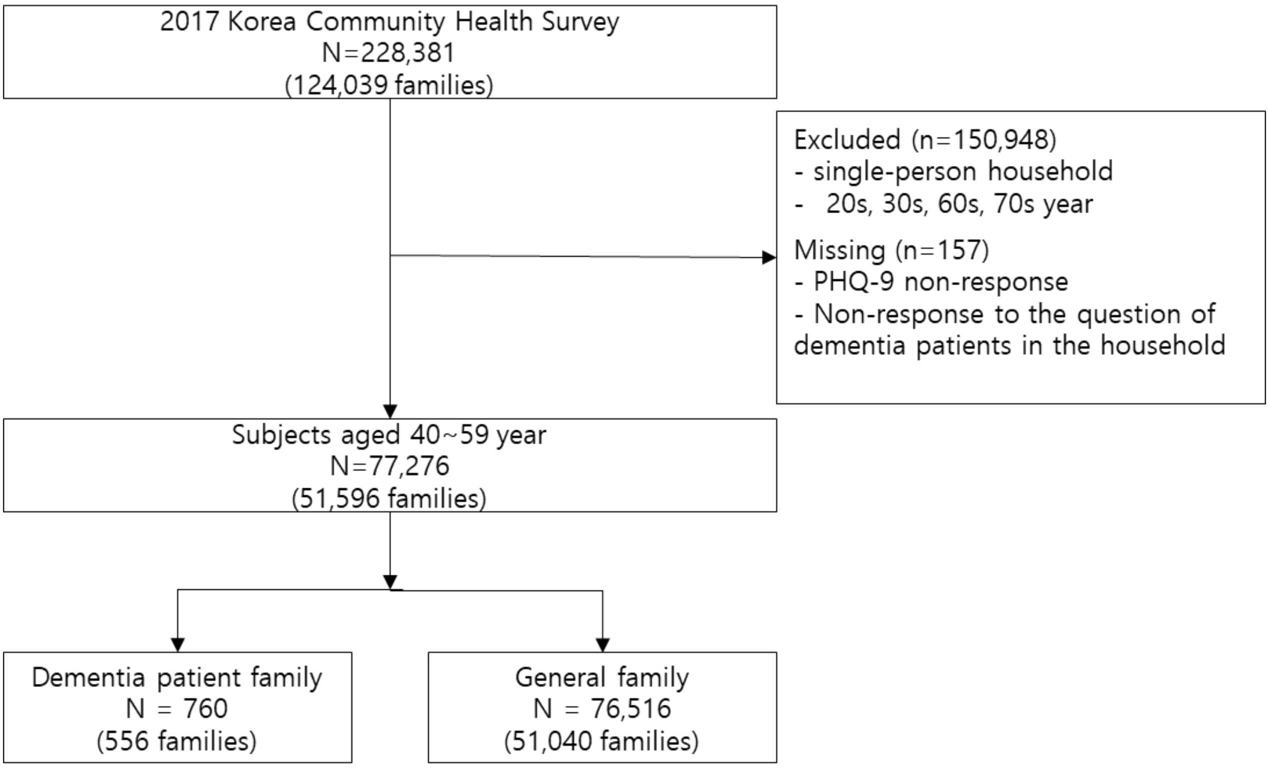

Figure 1. Flow chart of this study

Volume: 42, Article ID: e2020031
https://doi.org/10.4178/epih.e2020031

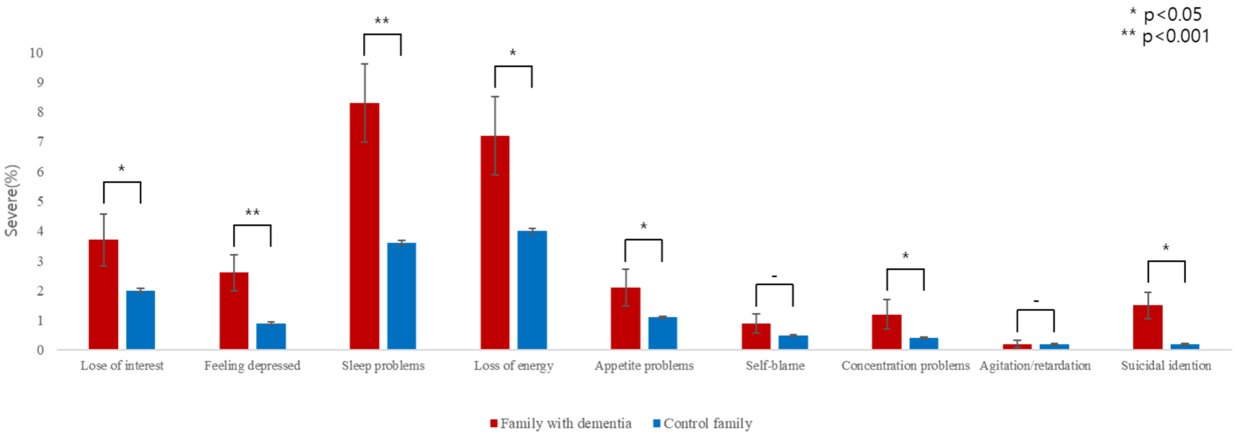

Figure 2. Proportion of severe answered PHQ-9 item between family with dementia patients at home and control family

Volume: 42, Article ID: e2020031
https://doi.org/10.4178/epih.e2020031

Appendix Table 1. Odds ratio for PHQ-9 detected depressive symptom between family with dementia patients at home and control family(age 40-59)

	Depressive symptom			
Age	Family with dementia	Control family	Crude OR	Adjusted OR
40-59	35 (4.4)	1,431 (1.9)	2.457 (1.557-3.878)	1.716 (1.032-2.853)

† Values are presented as number (%) or odds ratio (95% confidence interval).
‡ Adjusted for age, gender, household monthly income, employment, education, household by generation, town, current smoking and monthly alcohol drinking

Appendix Table 2. Odds ratio for PHQ-9 detected depressive symptom between family with dementia patients at home and control family(age 30-69)

	Depressive symptom			
Age	Family with dementia	Control family	Crude OR	Adjusted OR
30-69	65 (5.4)	2,964 (2.2)	2.560 (1.795-3.650)	1.813 (1.246-2.636)

† Values are presented as number (%) or odds ratio (95% confidence interval).
‡ Adjusted for age, gender, household monthly income, employment, education, household by generation, town, current smoking and monthly alcohol drinking

Appendix Table 3. Odds ratio for PHQ-9 detected depressive symptom between family with dementia patients at home and control family(age 40-69)

	Depressive symptom			
Age	Family with dementia	Control family	Crude OR	Adjusted OR
40-69	61 (5.8)	2,273 (2.0)	2.921 (2.022-4.220)	1.925 (1.303-2.845)

† Values are presented as number (%) or odds ratio (95% confidence interval).
‡ Adjusted for age, gender, household monthly income, employment, education, household by generation, town, current smoking and monthly alcohol drinking
